# Nonsecretory Multiple Myeloma Presenting as an Intestinal Tumor

**DOI:** 10.1155/2015/818715

**Published:** 2015-04-16

**Authors:** Diana Triantafyllopoulou, Stuart Mellor, Catherine Cargo, Ioannis Gkikas, Jagdish Adiyodi, Ayub Ali Bin, Neil Sahasrabudhe, Margaret Rokicka

**Affiliations:** ^1^Hematology Department, Royal Blackburn Hospital, Haslingden Road, Blackburn, Lancashire BB2 3HH, UK; ^2^Radiology Department, Royal Blackburn Hospital, Haslingden Road, Blackburn, Lancashire BB2 3HH, UK; ^3^HMDS, Leeds Cancer Center, Saint James's Institute of Oncology, Level 3, Bexley Wing, Leeds LS9 7TF, UK; ^4^Gastroenterology Department, Royal Blackburn Hospital, Haslingden Road, Blackburn, Lancashire BB2 3HH, UK; ^5^Pathology Department, Royal Blackburn Hospital, Haslingden Road, Blackburn, Lancashire BB2 3HH, UK

## Abstract

We report a case of a 43-year-old Caucasian man who presented with colicky abdominal pain and microcytic hypochromic anemia. The patient underwent a colonoscopy where a tumor was seen in the ascending colon; histology showed plasmacytoma of the colon. From the protein electrophoresis, no monoclonal band or free light chains were detected nor was urinary Bence Jones protein present. A bone marrow biopsy showed plasma cell myeloma. To the best of our knowledge, this is the first case of nonsecretory multiple myeloma presenting as plasmacytoma of the colon.

## 1. Introduction

Multiple myeloma is a malignant neoplasm of the bone marrow and accounts for approximately 10% of all hematologic malignancies, as discussed by da Silva et al. [1]. The tumor cells of this disease are plasma cells and produce immunoglobulins and/or light chains. Nonsecretory myeloma is characterised by the absence of an M protein in both the serum and urine occurring in approximately 2% of all patients with multiple myeloma. Patients with nonsecretory multiple myeloma (NSMM) are typically 10 years younger than in secretory myeloma. Anemia and increased serum calcium are much less common. Kidney failure is rare, probably due to the absence of monoclonal light chains (Bence Jones protein) in the urine. The diagnosis depends upon the demonstration of an excess of monoclonal (kappa or lambda) plasma cells in the bone marrow. The gastrointestinal tract is rarely involved in MM. The small intestine and stomach are the most common sites of spread and, rarely, the colon can be involved as discussed by Goldstein and Poker [2]. We describe a rare case of NSMM presenting as an intestinal colonic mass in a 43-year-old Caucasian man.

As intestinal plasmacytoma has not been reported in NSMM, we felt that this case was worth reporting.

## 2. Case Presentation

**Figure 1:**
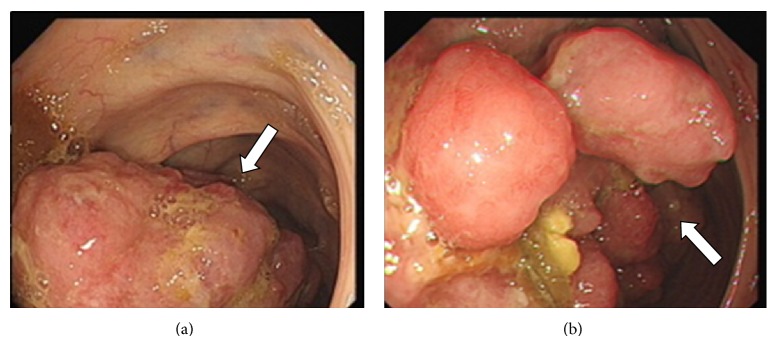
Endoscopic views of mass in ascending colon, occupying more than two thirds of the lumen. Proximal end (a) and central part of mass (b).

A 43-year-old man presented to the gastroenterology department with colicky abdominal pain, microcytic hypochromic anemia, and fatigue. At colonoscopy, a tumor was seen in the ascending colon, as shown in Figure 1.

His FBC was as follows: WBC: 17.7 × 109/L (normal: 4.0–11.0), NE: 15.9 × 109/L (normal: 2.0–7.5), LY: 0.7 × 109/L (normal: 1.5–4.0) MO: 0.8 × 109/L (normal: 0.2–0.8), HB: 98 g/L (normal: 130–180), MCV: 66.9 FL (normal: 76–100), MCH: 19.5 pg (normal: 27–32), MCHC: 291 g/L (normal: 310–360), RDW: 20 (normal: 10–15.7) crea: 198 *μ*mol/L (normal: 58–110), urea: 11.0 mmol/L (normal: 2.5–7.8), bili: 8 *μ*mol/L (normal: 0–21), ALT: 23 (normal: 3–53), ALP: 75 IU/L (normal: 30–130), albumin: 23 g/L (normal: 35–50), Ca: 2.45 mmol/L (normal: 2.20–2.60), and globulin: 20 g/L (normal: 18–36).

**Figure 2:**
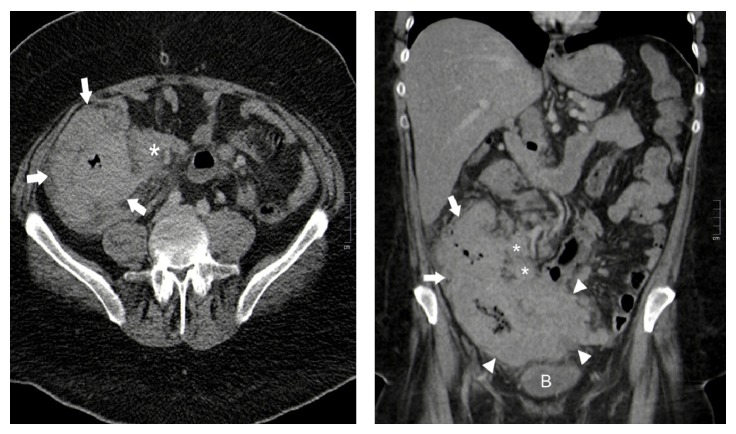
Axial and coronal CT images* through the abdomen and pelvis* from a portal venous phase whole body CT scan demonstrating marked diffuse thickening of the caecum (arrowheads) and ascending colon (arrows) with associated ileocolic lymphadenopathy (asterisks). Note the homogenous enhancement and texture of the colonic wall thickening, appearances which are more commonly seen with bowel lymphomas than bowel carcinomas. B = bladder.

A CT scan of the thorax, abdomen, and pelvis showed a large soft tissue mass extending from the right iliac fossa into the pelvis with involvement of the adjacent small bowel loops. There was extensive bowel wall thickening of the caecum and ascending colon as shown in Figure 2, extending over a length of approximately 20 cm, with the wall measuring up to 4.5 cm in thickness. Regional and mesenteric nodes were demonstrated and there was a suspicion of right juxtadiaphragmatic pulmonary and umbilical deposits. A subsequent dedicated skeletal survey was negative for lytic lesions.

CT scan pictures to follow as shown in Figure 2.

**Figure 3:**
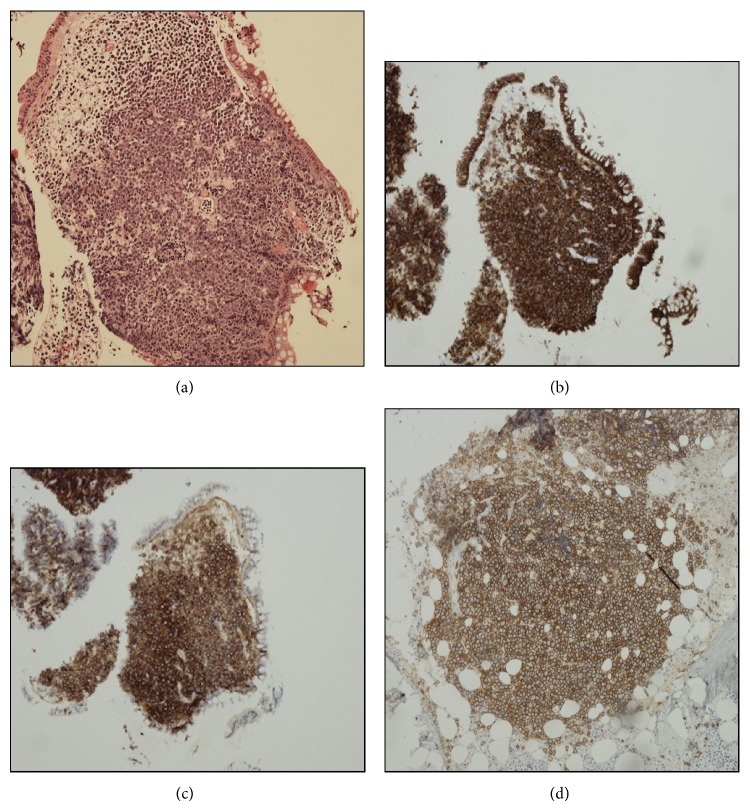
Histological assessment of the colonic mass and bone marrow trephine; (a) H&E ×10 showing diffuse infiltration of the colonic mucosa by large blastic cells with expression of CD138 (×4) (b) and CD56 (×4) (c). Focal infiltration by CD138 (×10) expressing blastic cells in the bone marrow trephine biopsy (d).

The fragments of colonic mucosa showed extensive infiltration by large cells with eccentric nuclei and prominent nucleoli as shown in Figure 3(a). Frequent mitotic figures were noted and the Ki67 proliferation index was 100%. The cells expressed plasma cell associated markers including CD138 and IRF4 as shown in Figure 3(b) though they lacked CD19 and showed strong expression of CD56 as shown in Figure 3(c). There was no evidence of EBV. FISH identified a MYC rearrangement. The differential included plasmablastic lymphoma or soft tissue plasmacytoma though the presence of strong CD56 and lack of EBV favoured the latter. A subsequent bone marrow biopsy confirmed the diagnosis of myeloma with neoplastic plasma cells identified by flow cytometry (CD19^−^CD56^++^CD27^−^CD45^−^) and a multifocal infiltrate of plasma cells identified with similar blastic morphology as shown in Figure 3(d). A c-MYC rearrangement was detected by FISH on the primary tissue biopsy and there was no evidence of other recurrent cytogenetic abnormalities.

The patient was then referred to the haematology department where serum protein electrophoresis was performed, but no monoclonal band was detected. IgG: 5.7 g/L (normal: 6–16), IgA: 2.5 g/L (normal: 0.80–4), IgM: <0.25 g/L (normal: 0.40–2.30), free kappa chains which were normal 15.6 mg/L (normal: 6.7–22.4), free lambda chains: 20.2 (normal: 8.3–27), SFLCR: 0.77 (normal: 0.31–1.56), within normal limits, and UBJ: negative, and urinary protein immunofixation did not detect a light chain band.

Consider the following: ESR: 47 mm/hr (normal: 3–15), ferritin: 55 *μ*g/L (normal: 30–365), CRP: 162 mg/L (normal: 0–10), and LDH: 1415 IU/L (normal: 313–618).

The patient tested negative for HIV and EBV.

The bone marrow biopsy showed a normocellular marrow with active trilineage hematopoiesis.

There was a focal infiltrate of large blastic cells and a neoplastic plasma cell population was identified by flow cytometry. The features were consistent with plasma cell myeloma.

From the flow cytometry the plasma cells were 1.8% of leucocytes, of which 90% had a neoplastic phenotype CD19^−^, CD56^++^, CD27^−^, and CD45^−^.

Unfortunately the patient died of a pulmonary embolism before treatment could be commenced. Postmortem examination showed extensive tumour deposits in the abdominal cavity. The appearance in the abdominal cavity was consistent with disseminated myelomatous involvement. The plasma cell myeloma had put an increased risk for the development of pulmonary embolism and therefore would have also contributed indirectly to the cause of death. Patients with newly diagnosed multiple myeloma, prior to having received therapy, seem to be at increased risk for venous thromboembolic disease and pulmonary embolism as discussed by Auwerda et al. [3].

## 3. Discussion

This is a very unusual case of NSMM presenting as extramedullary myeloma. In the absence of a paraprotein the diagnosis of blastic plasma cell myeloma and its distinction from plasmablastic lymphoma (PBL) is challenging as these malignancies share many similar features. On histological assessment features that favour a PBL would include partial retention of CD20, PAX5, and germinal centre markers along with weak/partial CD138 and evidence of EBV or a c-MYC rearrangement as discussed by Montes-Moreno et al. [4]. While the presence of a c-MYC rearrangement in this case favoured a PBL, these recurrent abnormalities are reported in myeloma as discussed by Affer et al. [5] and the demonstration of neoplastic plasma cells with a “typical” myeloma phenotype by flow cytometry in the bone marrow was more consistent with a blastic plasma cell myeloma.

Patients with NSMM are treated in the same fashion as MM; however kidney problems associated with myeloma are less common in NSMM. The lack of an M protein in the serum and urine in NSMM may be due to the inability of plasma cells to excrete the M protein, low synthetic capacity of M protein formation, degradation of the M protein within the plasma cell, or rapid degradation of M protein after secretion from the plasma cell. The diagnosis of NSMM depends upon the demonstration of monoclonal (kappa or lambda) plasma cells in the bone marrow. All the segments of the gastrointestinal tract may be involved by plasma cell infiltration. The small bowel is the most common site of involvement, followed by the stomach, colon, and esophagus, as discussed by Pimentel and Van Stolk [6].

Males in the age of 35–85 are more commonly affected with colon plasmacytoma.

The most common symptom is abdominal pain which may be accompanied by change in bowel habit and rectal bleeding. Endoscopically, it may appear as a discrete ulcer, an ulcerated mass, thickened mucosal folds, or polyps as discussed by Karam et al. [7]. Appearance varies and may be similar to other more common conditions, such as poorly differentiated or metastatic neoplasms, lymphoma (particularly MALT), and gastrointestinal amyloidosis as discussed by Telakis et al. [8]. Gastrointestinal plasmacytoma in the course of multiple myeloma is extremely rare accounting for approximately 0.9% as Talamo et al. showed in a large retrospective study conducted on 2.585 recruited myeloma patients [9].

Chemotherapy is the treatment of choice in cases of associated systemic disease, with resection if indicated. Radiotherapy is also an option, because plasmacytomas are known to be radiosensitive. Bladé et al. have reported the lack of efficacy of thalidomide on EMP, with progression of the extramedullary disease, despite good serological and medullary response. Bortezomib is reported to be efficacious in EMD, whereas there are no convincing data on the efficacy of lenalidomide, as discussed by Bladé et al. [10].

Two studies found that extramedullary disease is associated with shorter progression free survival and overall survival, even in the era of novel agents, as discussed by Varettoni and Wu [11, 12]. Unfortunately, prognosis remains very poor despite aggressive treatment.

## Abbreviations

NSMM: Nonsecretory multiple myeloma
M protein: Monoclonal protein
ALT: Alanine aminotransferase
LDH: Lactate dehydrogenase
EMD: Extramedullary disease
EMP: Extramedullary plasmacytoma
PBL: Plasmablastic lymphoma.
